# Multiconfigurational nature of the chemical bond in beryllium molecule

**DOI:** 10.1007/s00894-026-06812-6

**Published:** 2026-06-27

**Authors:** Berkay Sütay

**Affiliations:** Department of Chemistry, Istanbul Technical University, Istanbul, 34469 Turkey

**Keywords:** Beryllium molecule, Ab initio, Nondynamical correlation, Potential energy curve

## Abstract

**Context:**

Despite having only eight electrons, accurate prediction of the dissociation energy of the weakly bound beryllium molecule remains a stringent test for quantum chemical methods. Although its formal bond order is zero, Be_2_ exhibits an unusual bond that is stronger than dispersion forces yet weaker than typical chemical bonds, arising from the interplay of nondynamical and dynamical correlation effects. In this work, a detailed first-principles study of the ground state of Be_2_ is presented. An accurate ab initio potential energy curve (PEC) is computed and extrapolated to the complete basis set (CBS) limit. The contributions of nondynamical and dynamical correlation effects to the dissociation energy are rigorously analyzed within the Nonclosed Shell Many Electron Theory (NCMET) framework. NCMET(ND) predicted that the binding energy results predominantly from the nondynamical correlation, with a significant but smaller contribution from dynamical correlation. It is further found that the triple excitations play the crucial role in locating the correct minimum. An eight-parameter model potential is proposed, and new values for dispersion coefficients are reported. The inclusion of the C_10_ term was found essential to support the full set of twelve vibrational states observed experimentally. The vibrational states are obtained by solving the Lippmann–Schwinger equation in momentum space using the developed potential.

**Methods:**

An accurate ab initio PEC was computed using a range of quantum chemical methods, including Nonclosed Shell Many Electron Theory (NCMET), high-order perturbation theory (MP8 and MP9), and full CI in pentuple-zeta basis and extrapolated to the CBS limit. The nondynamical correlation energy of Be_2_ was calculated using NCMET(ND), including internal and semi-internal correlations, while dynamical correlation effects were described using second-order configuration interaction (SOCI) and near-NCMET wave functions. Multiconfigurational (MC) wave functions were constructed within the MCCI framework using CSFs under D_2h_ symmetry, with an active space of approximately thirty orbitals in frozen core approximation. Correlation-consistent augmented basis sets (aug-cc-pVXZ, X=3–5) were employed. Core correlation effects were estimated using all-electron coupled-cluster singles, doubles, and noniterative triples (CCSD(T)) method with aug-cc-pCVXZ basis sets. The relativistic effects and the diagonal Born–Oppenheimer correction (DBOC) were included. Dissociation energy was calculated using a supermolecular approach at a large separation to ensure size consistency. All NCMET(ND), SOCI, near-NCMET, full CI, and relativistic calculations were performed in MOLPRO, while multireference coupled cluster (Mk-MRCC), symmetry-adapted perturbation theory (SAPT), and MPn calculations were carried out in PSI4.

**Supplementary Information:**

The online version contains supplementary material available at 10.1007/s00894-026-06812-6.

## Introduction

The beryllium molecule is one of the most frequently used benchmark systems in state-of-the-art theoretical chemistry. Despite numerous experimental and theoretical studies, the nature of bonding in Be_2_ remains not fully understood. The main difficulty in describing this notoriously complex bond stems from the different behavior of the wave function in the minimum and dissociation regions of the PEC, where even small energy errors may lead to qualitatively incorrect conclusions. A key feature is that the PEC at the Hartree–Fock level is strictly repulsive and exhibits UHF instability due to the near-degeneracy of the valence orbitals of beryllium atom. Although many methods that yield accurate results for other molecules have been applied to Be_2_, their predictions vary significantly with respect to the energy minimum: some indicate a van der Waals-like minimum, whereas others predict a slightly deeper minimum at shorter internuclear distances. While the equilibrium bond length (*R*_e_) correlates more closely with that of van der Waals molecules, the dissociation energy is more comparable to that of covalently bonded molecules (see excellent review, ref [1]). The nature of the bonding in diberyllium is quite uncommon [2–4] and remains a considerable challenge in quantum chemistry. The small 2s-2p gap in atomic beryllium, together with an avoided crossing between 2s^2^ + 2s^2^ and 2s2p_z_ + 2s2p_z_ curves in the presence of internal correlation effects, leads to a bound system. Consequently, a multiconfigurational (MC) description of the PEC is required to obtain a qualitatively correct chemical picture. The most recent experimental study (2014) [5, 6] triggered renewed interest in Be_2_, reporting all 12 vibrational levels. The best estimates of *R*_e_ and binding energy (D_e_) values were predicted to be 2.45 Å and 935 cm^−1^, respectively, and a 12th level was also proposed [7]. At shorter internuclear distances, the interaction is covalent-like, whereas at larger separations it is dominated by van der Waals forces [8, 9].

The properties of the beryllium molecule are highly sensitive to electron correlation and basis set quality. Various coupled-cluster approaches have been applied to reproduce the PEC of Be_2_ [10–13]. Spirko [14] reported a lower binding energy (923 cm^−1^) using MRCI. A valence complete active space type diffusion quantum Monte Carlo (valCAS-DMC) study with 16 orbitals yielded 846 cm^−1^ [15], with additional QMC results reported elsewhere [16, 17]. Gdanitz [18] obtained 902 cm^−1^ value using the r_12_-MR(CAS)-ACPF method, while Schmidt et al. [19] predicted 846 cm^−1^ from valence CAS self-consistent field (valCASSCF) with full CI corrections. Lesiuk et al. [20] reported an extrapolated value of 863 cm^−1^.

In the present work, the multiconfigurational nature of Be_2_ is examined in great detail through ab initio ground-state PEC at the complete basis set (CBS) limit, with full inclusion of correlation effects. A range of methods were employed, including multireference approaches (second-order CI (SOCI) and near-NCMET), many-body perturbation theory (MP6 and MP8), and MCCI-based methods (NCMET(ND) and CASCI). The effect of nondynamical and dynamical correlation effects were systematically analyzed. Dispersion coefficients up to C_12_ were predicted, and all 12 vibrational levels were supported by the proposed eight-parameter potential energy function.

## Computational methodology

Given Φ_0_ as the HF determinant (with H_0_Φ_0_ = E_0_Φ_0_ and H = H_0_ + H’), then the energy may be written as the sum of E_HF_ and the corrections starting from second order of perturbation. If the exact wave function is expressed as the sum of HF determinant and the correlation function (χ), i.e., Ψ = Φ_0_ + χ, then the energy is calculated via variational principle (Eq. 1).

1 $$E\leq\frac{\left\langle\Psi\left|H\right|\Psi\right\rangle}{\left\langle\Psi\left|\Psi\right.\right\rangle} = \begin{array}{c}E_{HF}+\frac{2\left\langle\Phi_0\left|H\right|\chi\right\rangle+\left\langle\chi\left|H\right|\chi\right\rangle}{1+\left\langle\chi\left|\chi\right.\right\rangle}\\ E_{HF}+\frac{2\left\langle\Phi_0\left|H'-E^{(1)}\right|\chi\right\rangle+\left\langle\chi\left|H_0-E_0\right|\chi\right\rangle+\left\langle\chi\left|H'-E^{(1)}\right|\chi\right\rangle}{1+\left\langle\chi\left|\chi\right.\right\rangle}\end{array}$$

Here, the first term in numerator gives E^(2)^ for χ_1_, which accounts for the lion’s share of the correlation energy from double excitations. The second term corresponds to the other dominant terms such as disconnected quadruples, within E^(4)^, E^(6)^, E^(8)^, … perturbation contributions. The last term relates to the presence of other effects, such as coupling terms, within E^(3)^, E^(5)^, E^(7)^, E^(9)^, etc. Finally, the large portion in the numerator is varied to include the dominant excitation types, which cancel out much of the integral term in the denominator. In the present work, the nondynamical correlation energy of Be_2_ molecule was calculated using NCMET(ND) to include all nondynamical correlation terms; i.e., the sum of internal and semi-internal correlations. NCMET(ND) and near-NCMET variants of the theory were applied to clarify the contributions of nondynamical and dynamical correlation into valence correlation energy and the dissociation energy. A full CI calculation in the valence active space is called valence CASSCF (valCASSCF) which includes the internal (static) correlation energy. Semi-internal correlation, together with the polarization effects, is accounted for by configurations constructed from the orbitals of the valence space together with a correlation orbital within the framework of the Nonclosed Shell Many Electron Theory (NCMET) of Sinanoğlu [21, 22], it is coupled to orbital spin- and symmetry-polarization effects. The sum of internal and semi-internal correlations corresponds to the nondynamical correlation energy [23]. The semi-internal correlation is also well-known and essentially used in excited state calculations (for example, see the Gaussian 09 manual [24]), its inclusion in MRPT and MRCI calculations is crucial [25, 26]. As noted by Sinanoğlu, internal correlation comprises excitations occurring entirely within the active-space orbitals, whereas semi-internal correlation involves at least one excitation from the active space to the virtual orbital space. A similar distinction has been introduced within the coupled-cluster framework [27]. The dynamical (external) correlation is then described by the configurations in which two or more electrons are excited out of the valence active space. The multiconfigurational NCMET(ND) wave function is generated within a space spanned by a small set of CSFs in MCCI formalism under spin and symmetry restrictions. The CSFs were constructed under D_2h_ abelian point-group symmetry. The active space was set to a space of almost thirty orbitals to let the semi-internal correlation converge. All relevant nondynamical (ND) type triple and quadruple excitation terms (internal and semi-internal triples and quadruples) were included in NCMET(ND) wave function to support a well-dressed PEC. The core electrons were kept frozen. The augmented version of the correlation consistent polarized valence basis sets (aug-cc-pVXZ) was used. Dynamical part of high-order correlations were included in second-order CI (SOCI) and near-full NCMET wave functions. All possible single and double excitations from the NCMET(ND) reference wave function were included in the near-NCMET wave function. CBS extrapolation was used to reduce the basis set truncation error, using the aug-cc-pVXZ (X: 3, 4, 5) basis set hierarchy (see Supp. Info-part A).

Supermolecular approach was applied in the calculation of dissociation energy to overcome the size-consistency problem, by replacing the separated atom limit with a stretched geometry (50 Å). The quality of the wave function was assessed using the non-parallelity error (NPE) and the size-consistency error (SCE). The correction terms beyond nonrelativistic hamiltonian in infinite nuclear mass and frozen-core approximation (NR, FC) were subtracted from the experimental dissociation energy to make a reasonable comparison between theory and experiment. The effects of core correlation were estimated from all-electron CCSD(T) calculations using augmented core- and valence-polarized triple and quadruple-zeta basis sets (aug-cc-pCVXZ), then by using the two-point X^−3^ type extrapolation formula. Scalar relativistic effects were calculated using Douglas–Kroll–Hess transformation. An uncontracted quadruple-zeta basis was employed for the large-component of the spinor and the corresponding small-component basis functions were generated using the kinetic balance. The contribution of diagonal Born–Oppenheimer correction (DBOC), Breit terms and quantum electrodynamics (QED) corrections to binding energy were all found negligible (see Supp. Info- part B). The Dirac–Coulomb–Breit (DCB) calculations were performed with the speed of light, c = 137.035999139, for a point nucleus. NCMET(ND), SOCI, near-NCMET, full CI calculations and relativistic corrections were performed in MOLPRO [28]. The symmetry adapted perturbation theory (SAPT) [29, 30], Møller–Plesset perturbation theory (MPn at 6th [31] to 8th order [32]) and CISDT computations were performed in PSI4 suite [33]. The NCMET(ND) wave function is available in Supp. Info-part E. Orbital relaxation effects were already included in SAPT calculations. All calculations were performed on a high-performance computing cluster.

## Results and discussion

Beryllium molecule is simple in structure, yet it presents significant challenges for ab initio methods. The long range attractive force is mostly dynamical correlation and may be considered as dispersion interaction. Around equilibrium separation, the orbital overlap is strong which leads to hybridization in RHF and also internal correlation effects. The HF method gives − 2586 cm^−1^ binding energy; in other words, it predicts a fully repulsive interaction. Both the T_1_ diagnostic and the largest T_2_ amplitude calculated at the CCSD(T)/QZ level of theory were found to be on the order of 0.02. While the T_1_ diagnostic by itself is not sufficient to establish multireference character [34], the magnitude of the largest T_2_ amplitude supports the quasi-degenerate nature of the Be_2_ molecule.

Table 1 shows the total (NR,FC) electronic energies of beryllium molecule in infinite nuclear mass approximation, calculated by different levels of theories. HF and valCASCI methods give a completely repulsive interaction; however, NCMET(ND) predicts a very shallow minimum at 4.5 Å. By adding the dominant external double excitations (almost 150 external terms), the minimum was still found beyond 2.5 Å separation. This type of MCCI calculation gives a positive binding energy compared to valCASCI, while did not improve the binding energy compared to NCMET(ND). This may be attributed to the necessity of triple excitations.

**Table 1:** Total (NR,FC) electronic energy of ^∞^Be_2_ in QZ basis ^a^

Method	R_e_ (Å)	E_e_ (a.u.)	D_e_ (cm^−1^)
NCMET(ND)	4.50	− 29.19896	- d
250-MCCI^b,c^	4.15	− 29.20516	38.5
CASCI^b^	2.85	− 29.23215	678.6
SOCI	2.45	− 29.23774	570.2
Near NCMET	2.45	− 29.23986	601.7
Full CI	2.45	− 29.23987	605.2

^a ^HF energy: − 29.13406 a.u., valCASCI energy: − 29.16645 a.u., ^b^ active space with 30 orbitals, see Supp. Info-part E. ^c^ NCMET(ND) + 150 external correlation terms. ^d^ repulsive at R_e_, but very shallow at 4.5 Å

**Fig. 1:**
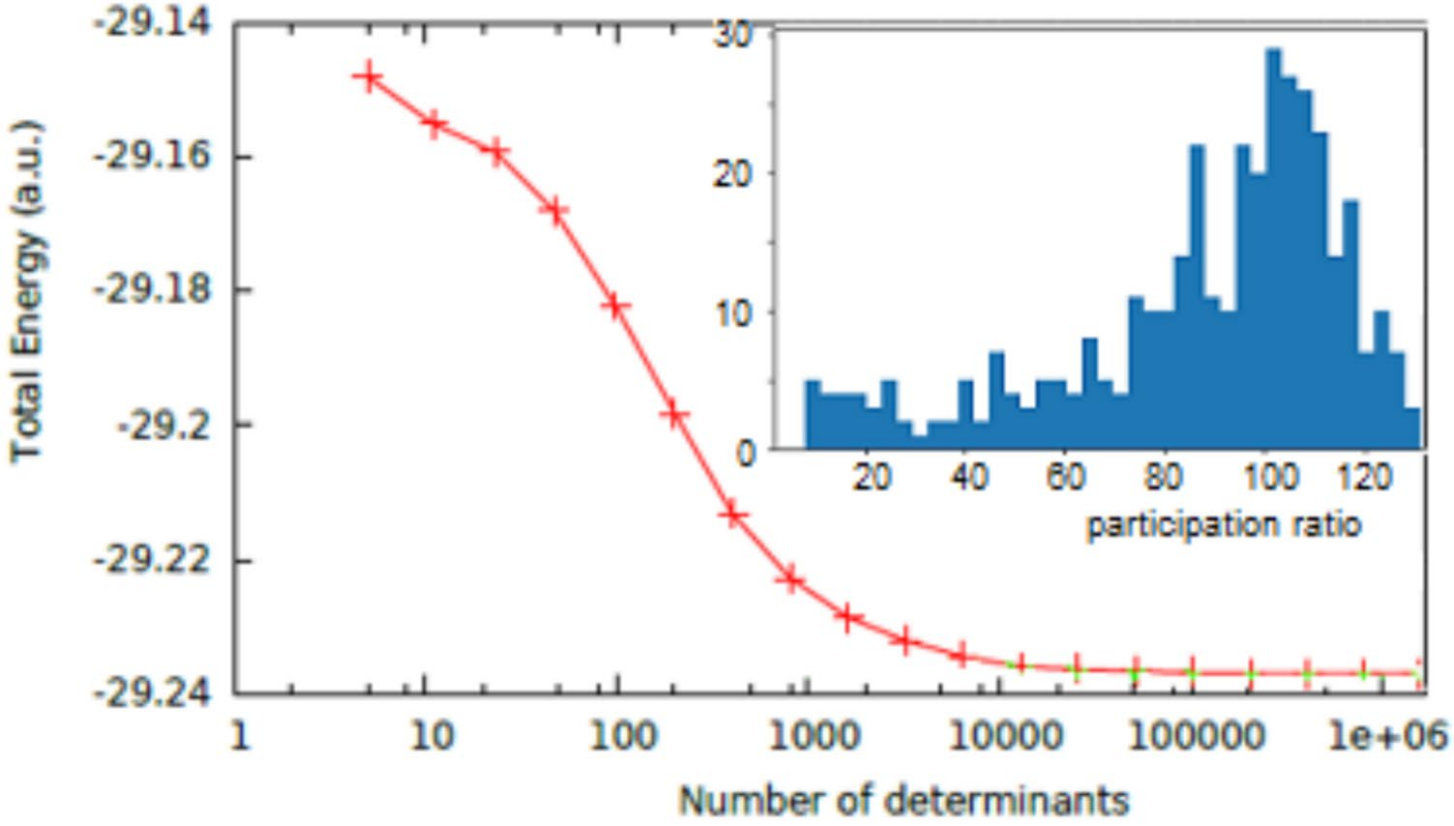
The convergence of full CI energy of ^∞^Be_2_ in QZ basis (the histogram shows how spread out the eigenvectors in determinant space, that is, the most participation ratio values lie between 80 and 120, indicating the high multireference character of Be_2_)

The 250-MCCI wave function obtained by augmenting the NCMET(ND) wave function, which already includes nondynamical excitations up to quadruples, with the most dominant double excitations was able to shift the minimum only from 4.5 to 4.15 Å. In contrast, CASCI calculations performed within an active space of the same size, which also include a significant portion of external triples, shifted the minimum to as low as 2.85 Å. The reason for that is the unbalanced inclusion of higher correlation terms and the absence of large number of triples and quadruples out of the active space. These results indicate that triple excitations play the crucial role in locating the correct minimum. However, the most dominant triples alone -those originating from orbitals closer in energy to the valence space are not sufficient. Instead, all triples accessible within the orbital basis set must be taken into account. SOCI, near-NCMET and full CI methods all predicted the minimum at 2.45 Å. The convergence of the full CI energy of beryllium molecule, at equilibrium geometry, as a function of the number of determinants, was shown in Fig. 1.

The valence correlation energy of ground-state ^∞^Be_2_ at its equilibrium geometry was calculated at different levels of MR theories, Table 2. After the addition of the corrections beyond BO approximation to the nonrelativistic total electronic energy of ^∞^Be_2_, the exact (experimental) total energy of the molecule was estimated to be − 29.34114 a.u. (see Supporting Information-part B). In the present work, TQ contribution to valence correlation energy was found to be −0.01093 a.u. (T ~ − 0.0041 a.u., Q ~ − 0.0068) which corresponds to 10% of the valence correlation. The contributions of nondynamical SD and TQ correlations were estimated about − 0.0633 a.u. and − 0.0016 a.u., respectively.

**Table 2:** The valence correlation energy of ^∞^Be_2_ (a.u.) at R_e_ in CBS(angular) limit ^a^

Method	E_e_	E_corr_	D_e_ (cm^−1^)
SOCI	− 29.24023	− 0.1060	797.6
MP6	− 29.24069	− 0.1064	825.5
Near NCMET	− 29.24194	− 0.1077	843.1
MP8	− 29.24221	− 0.1080	850.0
Full CI	− 29.24238 *SD* *TQ*	− 0.1081 − *0.09717* − *0.01093*	864.0

^a ^HF limit is − 29.13428 a.u

The binding energy of the molecule was also calculated at higher levels of theory and summarized in Table 2. The SOCI method predicts the binding energy as 769 cm^−1^ in aug-cc-pVQZ basis and 797.6 cm^−1^ in CBS limit. The dissociation limit (R_0_) of the molecule was estimated to be 7 Å. As shown in Table 2, all applied methods converge to an equilibrium bond length of 2.45 Å (to two decimal places) at the CBS limit. All electron full CI energy was also estimated to be − 29.3390 a.u. which gives an all-electron correlation energy as − 0.2048 a.u., and the binding energy as 937 cm^−1^ (adding corrections beyond BO approximation gives a final value of 934 cm^−1^).

It is obvious that the quadruples also make a noticeable contribution to the valence correlation energy [4]. Full CI result, equivalent to CCSDTQ [35] within the frozen-core approximation, predicts a binding energy of 814 cm^−1^ in the aug-cc-pVQZ basis and 864 cm^−1^ in CBS limit. Upon separating the valence correlation energy of the ground state ^∞^Be_2_ into nondynamical (ND) and dynamical (D) correlation, the nondynamical correlation energy was predicted to be − 0.0650 a.u. from the NCMET(ND) calculation at equilibrium geometry. The contributions of internal and semi-internal correlation to nondynamical correlation were calculated to be − 0.0319 a.u. and − 0.0331 a.u. respectively, Table 3.

**Table 3:** The separation of valence correlation effects in ^∞^Be_2_

	ND	D	Valence corr. energy
R_e_ = 2.45 Å	− 0.0650 a.u *SD* = − *0.0633* *TQ* = − *0.0016*	− 0.0431 a.u *SD* = − *0.0339* *TQ* = − *0.0093*	− 0.1081 a.u *SD* = − *0.0972* *TQ* = − *0.0109*
50 Å	− 0.0749	− 0.0489	− 0.1238 a.u
	ΔHF	Δcorrelation	D_e_ (cm^−1^)
NCMET(ND)	− 2621	2195 Δint = 353 Δsemi-int = 1842	-
Full CI	− 2586	3450 ΔND = 2195 ΔD = 1255	864

The NCMET(ND) method predicts a repulsive potential at R_e_ separation. However, it predicts a bound state with a shallow potential well around 4.5 Å due to the lack of dynamical-type triple excitations that would support bonding at the correct *R* value. It is clear that nondynamical correlation alone is insufficient to explain bonding in the Be₂ molecule at the correct R distance. However, it is important to note that the reason for predicting the correct geometry is not a result of dynamical correlation as a whole, but rather of the presence of dynamical-type triple correlations. The contribution of ND and D-type correlation effects may be estimated as 2195 cm^−1^ and 1255 cm^−1^ respectively, which gives a total 3450 cm^−1^ correlation contribution to the D_e_(NR,FC) binding energy (864 cm^−1^). The contribution of internal correlation well agrees with the predictions of Dunning et al. [36] and Schmidt et al. [19]. The contribution of nondynamical correlation to D_e_ is almost twice of the dynamical correlation, however, it cannot indicate the ground state energy minimum alone and the dynamical correlation effects, specifically the external triples, are requisite to explain the binding in Be_2_ molecule. A further decomposition of binding energy was summarized in Table 4.

**Table 4:** The decomposition of binding energy in terms of different effects in detail (cm^−1^)

	ΔHF = − 2586	
ΔND = 2195 ΔD = 1255	** ← **Δcorrelation = 3450 →	Δ(T_1_ + T_2_ + T_2_^2^) = 2690 ΔT_3_ = 680 ΔT_4_ = 80
	***total NR, FC*** = 864	
	Δcore = 73.7	
	Δ(beyond BO app.) = − 3.7	
	Total = 934	

**Fig. 2:**
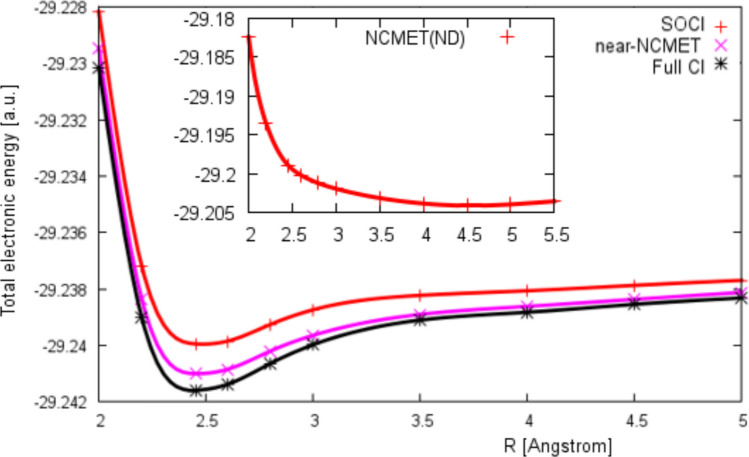
The PEC of ^∞^Be_2_ calculated at different levels of theories in aug-cc-pVQZ basis

The different minima found by the NCMET(ND) and SOCI methods were shown in Fig. 2. The double excitation term (2σ_u_)^2^ → (3σ_g_)^2^, which is a part of internal correlation, contributes to the NCMET(ND) wave function with a large weight. Compared to SOCI, the near-NCMET predicts a deeper minimum, namely 811 cm^−1^ with the aug-cc-pVQZ basis set and 843 cm^−1^ at the CBS limit. In other words, it accounts for 99% of the valence contribution to the NR, FC binding energy and reproduces 99.6% of the valence correlation energy. The near-NCMET PEC is found parallel to the full CI curve, Fig. 2, with a non-parallelity error (NPE) of 0.017 kcal mol^−1^.

**Fig. 3:**
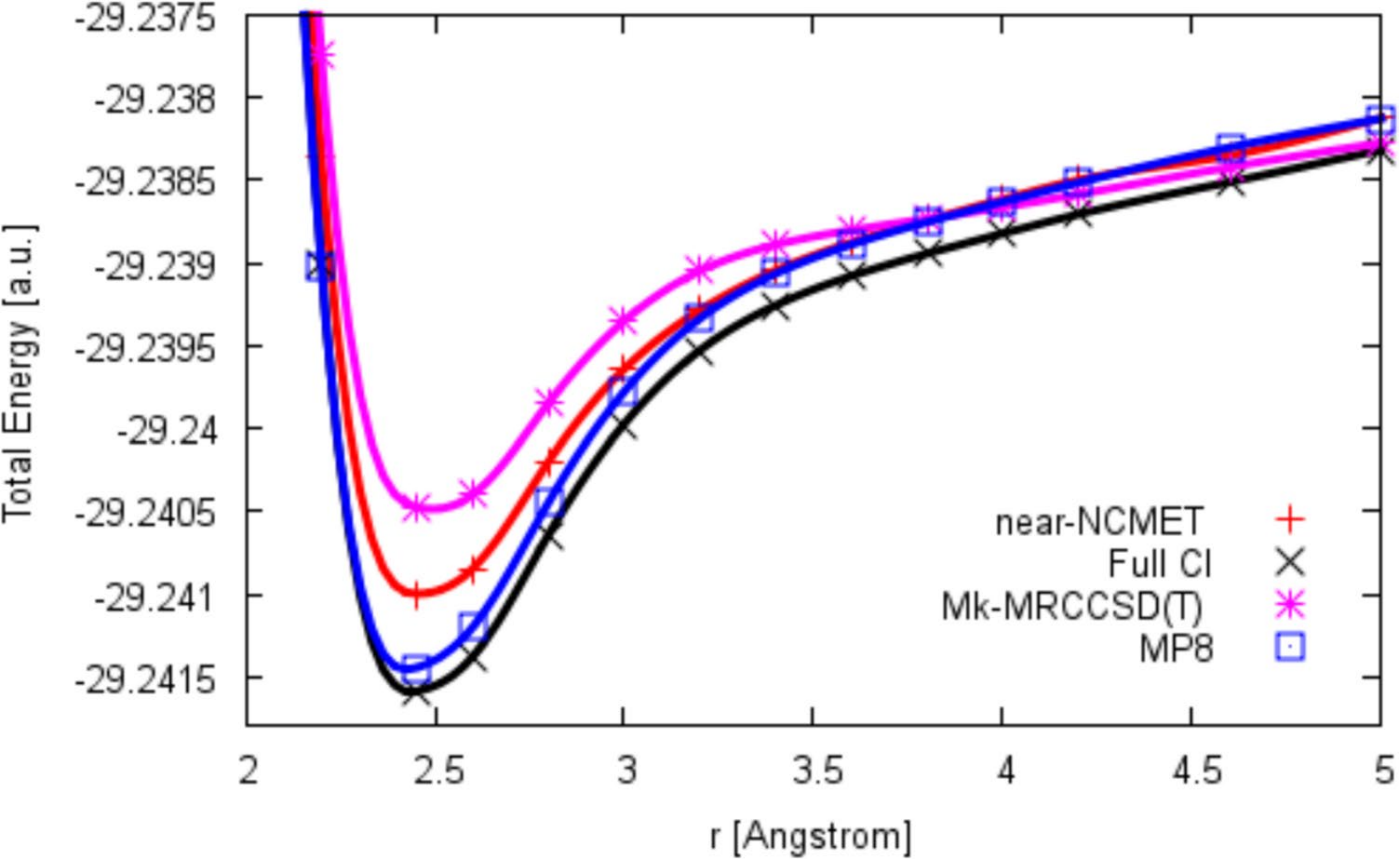
The PEC of ^∞^Be_2_ calculated by different levels of theories in aug-cc-pVQZ basis

The near-NCMET method predicts the correct asymptotic behavior at the dissociation region and gives a PEC parallel to full CI, Fig. 2. The frozen core full CI energy of ^∞^Be_2_ molecule at R_e_ is equal to − 29.24159 a.u. in QZ basis, almost equal to − 29.24158 a.u. from frozen core MRCISD calculation [37]. After the addition of core correlation, the energy is found to be − 29.3366 a.u., compared to − 29.3301 a.u. [38] and − 29.3336 a.u. [39] calculated by diffusion Monte Carlo (DMC) method and − 29.3377 a.u. [40] by CCSD(T) at CBS limit. The exact nonrelativistic total electronic energy of beryllium molecule is estimated to be − 29.3390 a.u. in this work. Finally, the higher level of theories, such as MP8 and near-NCMET, were used to compute the PEC of beryllium molecule and compared to full CI curve in aug-cc-pVQZ basis, Fig. 3 (see also Supporting Information-part D).

The strength of bonding in Be_2_ is weak by average chemical standards, but it is much stronger than the dispersion interaction with a somewhat deeper potential well. To understand the details of this interaction in terms of its components, the SAPT calculations were also performed, Table 5.

**Table 5:** SAPT energy components of ^∞^Be_2_ in QZ basis (kcal/mol) (the values in parantheses indicate the order in interaction potential and in the sum of fluctuation potentials of monomers)

Component	Contribution	Component	Contribution
Elst(10) Elst(12)	− 18.4431 0.6862	Exch-Ind(20) Exch-Ind(22) δHF(2)	22.7343 − 0.8168 − 2.6390
Exch(10) Exch(11) Exch(12)	43.0691 3.8531 − 2.6847		
		Disp(20) Disp(21) Disp(22) Exch-Disp(20)	− 11.6960 − 1.6847 0.1617 0.9539
Ind(20) Ind(22)	− 37.2302 1.3376		
		*Total*	− **2.4**

**Fig. 4:**
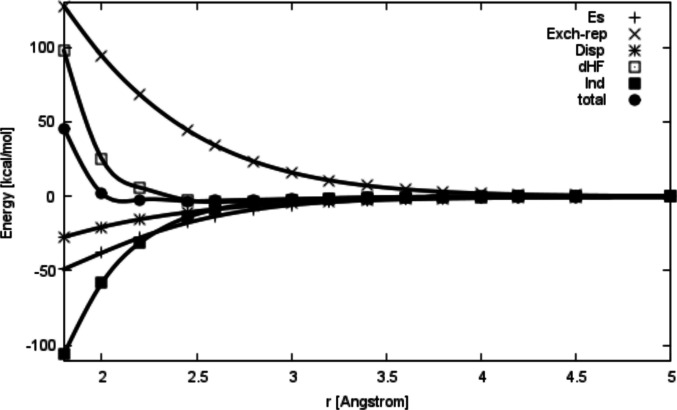
The potential energy profile of SAPT energy components of ^∞^Be_2_

The electrostatic and induction components equally contribute to the binding energy and are predicted to be − 17.7 and − 16.6 kcal/mol respectively. The dispersion component was found slightly weaker, which is almost − 12.3 kcal/mol, then one finds a total attraction in the order of − 46.6 kcal/mol. However, 44.2 kcal/mol exchange interaction cancels much of this attraction and gives a total binding energy as − 2.4 kcal/mol (especially, the induction interaction was significantly canceled by its exchange counterpart). The binding energy was also calculated as − 3.1 kcal/mol in aug-cc-pVQZ basis, which is comparable to − 3.3 kcal/mol value by Szalewicz et al. [13]. The potential energy profile of SAPT energy components was shown in Fig. 4.

The dispersion forces drive the interaction of two beryllium atoms at long range since the electrostatic and induction effects exponentially decay at larger distances. In contrast to weakly bonded noble gas dimers where the dispersion forces dominate, the electrostatic and induction effects were found stronger in covalently bonded Be_2_ molecule (net ES: − 17.8 kcal/mol, net ind: − 16.6 kcal/mol, net disp: − 12.3 kcal/mol).

The theoretical potential values were obtained using V(R) - V(50 Å) formula in an effort to explore a new model potential for Be_2_ molecule (Table 6). These values are comparable to the analytical potential which was fitted to the experimental data [6]. The dispersion interaction between two beryllium atoms in the long range part of the PEC may be approximated with -C_6_R^−6^-C_8_R^−8^-C_10_R^−10^ expansion. After omitting the C_10_ term in the dispersion expansion, it turns into a linear graph by setting C_8_ to the slope and C_6_ to the intercept. Then by fitting the full CI/5Z potential data, C_6_ and C_8_ coefficients were predicted to be 229 au.bohr^6^ and 10,659 au.bohr^8^ respectively (The values of V(R) at R = 6.5, 7, 8, 9 and 10 Å were used in fitting). The dispersion coefficients from the calculated atomic polarizabilities have been reported as C_6_ = 214 ± 3 and C_8_ = 10,230 ± 60 a.u. [41] which are consistent with our present data. Our most accurate FCI potential has the ability to recover long range dispersion interactions and clearly demonstrates that the correlation effects governing binding at the equilibrium distance are fundamentally different in nature. The experimental determination of these coefficients for the Be₂ molecule is still an important task. Therefore, the analytical potential values in Table 6 should not be considered as the direct reference values, but were used here solely for comparison.

**Table 6:** The calculated long range potential and the vibrational states of ^∞^Be_2_ (cm^−1^)

R (Å)	Full CI AV5Z ^a^	Analytical potential [19]	ref. [7]	v	E_v_	E_v_ (ref [19])
4.0	− 201.324	− 199.625	− 203.79	0	0	0
4.5	− 137.938	− 135.447	− 138.52	1	222.1	222.6
5.0	− 87.461	− 85.306	− 87.50	2	398.2	397.1
5.5	− 53.159	− 50.891	− 53.08	3	516.2	518.1
6.0	− 31.916	− 29.401	− 31.89	4	598.0	594.8
6.5	− 19.353	− 16.689	− 19.37	5	651.9	651.5
7.0	− 12.034	− 9.384	− 12.03	6	698.7	698.8
8.0	− 5.076	− 2.931	− 5.03	7	736.5	737.7
9.0	− 2.399	− 0.910	− 2.34	8	767.2	768.2
10.0	− 1.244	− 0.282	− 1.20	9	790.7	789.9
C_6_^a^	229	-	-	10	801.6	802.6
C_8_^a^	10,659	-	-	11	804.9	-

^a^1 au.bohr^6^ = 4809.7 cm^−1^Å^6^, 1 au.bohr^8^ = 1346 cm^−1^Å^8^, 1 au.bohr^10^ = 376.7 cm^−1^Å^10^

The calculated data was further fitted to a modified Tang-Toennies potential [42] which is a sum of Born–Mayer type short range repulsion term, van der Waals type long range attraction term and a correction term for mid-range potential (Eq. 2).

2 $$V(R)=V_{BM}+V_{vdW}+V_{correction}=Ae^{-bR}-\sum_{n=3}^5\left(1-e^{-bR}\sum_{k=0}^{2n}\frac{{(bR)}^k}{k!}\cdot\right)\frac{C_{2n}}{R^{2n}}+De^{(-gR-fR^2)}$$

By using our predetermined dispersion coefficients as C_6_ = 229 a.u. and C_8_ = 10,659 a.u., and applying Levenberg–Marquardt optimization with the estimated potential data points in Table 5, the optimized parameters were found to be A = 23.00, b = 1.26, D = − 4.30, g = 0.576857, f = 0.081859 (all in a.u.). Here, it must be remarked that the optimization was found highly sensitive to the values of D, g, and f parameters; among these, the minimum is strictly dependent on g and f parameters. This is because the Tang–Toennies potential gives a potential which has a minimum around 4.5 Å and the correct minimum may only be found after adding the last term in Eq. 2. This term is essential to correct the HF potential at long range [43]. Using this model potential, the scattering length (a_s_) for Be + Be scattering was estimated to be 1.25 Å (see Supporting Information-part C). This result is found to be closer to the value reported in ref [19] rather than to the scattering length value commonly estimated to be around 5 Å (a recent study predicted a lower value around 3.3 Å [44]).

To test the proposed model potential, the vibrational states of ground-state beryllium molecule were calculated (Table 6) by numerically solving the Lippmann–Schwinger equation in momentum space (Eq. 3).

3 $$\psi(p)=\frac1{E-{\displaystyle\frac{p^2}{2\mu}}}\int_0^\infty dp'p'^2V_0\left(p,p'\right)\psi\left(p'\right)$$

A finite momentum grid was employed, spanning up to a maximum momentum value. The grid was chosen to cover the relevant region of the spectrum, ensuring sufficient resolution for capturing bound states. The results obtained are in good agreement with those presented in reference [19], as reflected by an RMSE value of 1.5 cm^−1^. The elusive v = 11 bound state was also supported and all twelve vibrational levels were found close to the experimental data. To support the eleventh vibrational state, the presence of C_10_ coefficient was found significant and it was predicted to be 593,175 a.u. in this work. These energy levels were fitted to the Dunham expansion, Eq. 4. For vibrational motion only, J = 0.

4 $$ \begin{aligned}E_{v,J}&=\underset{i,j}{\sum} Y_{ij}\left(v+{\textstyle\frac12}\right)^i\\&=Y_{00}+Y_{10}\left(v+{\textstyle\frac12}\right)+Y_{20}\left(v+{\textstyle\frac12}\right)^2+Y_{30}\left(v+{\textstyle\frac12}\right)^3+Y_{40}\left(v+{\textstyle\frac12}\right)^4+\cdots\\ &=\omega_e\left(v+{\textstyle\frac12}\right)-\omega_ex_e\left(v+{\textstyle\frac12}\right)^2+\omega_ey_e\left(v+{\textstyle\frac12}\right)^3-\omega_ez_e\left(v+{\textstyle\frac12}\right)^4+\cdots\end{aligned}$$

A linear fit to level spacings of the vibrational energies using Dunham expansion yields the spectroscopic constants: ω_e_ = 284 cm^−1^, ω_e_x_e_ = 17.3 cm^−1^, ω_e_y_e_ = –0.67 cm^−1^ and ω_e_z_e_ = –0.02 cm^−1^, in good agreement with the parameters reported in ref [45].

## Conclusion

In this study, the ground-state potential energy curve (PEC) of the beryllium molecule was investigated using a wide range of high-level MC- and MR-type ab initio methods at CBS limit. A particular focus was given to the decomposition of the valence correlation energy into its nondynamical and dynamical components using the framework of NCMET(ND) level of theory. The valence correlation energy at equilibrium structure was predicted to be − 0.1081 a.u., decreasing to − 0.1232 a.u. at dissociation limit. The NCMET(ND) method, which explicitly includes all internal and semi-internal correlations, predicts a shallow minimum at long range (~ 4.5 Å) and yields a bound system. NCMET(ND) predicted that the binding energy results predominantly from the nondynamical correlation (2195 cm^−1^), with a significant but *smaller* contribution from dynamical correlation (1255 cm^−1^), yielding a total of 3450 cm^−1^ correlation energy contribution to the binding. It is clear that approximately 65% of the correlation binding originates from nondynamical correlation. However, nondynamical correlation alone is insufficient to describe the correct equilibrium internuclear separation in Be₂.

The effect of dynamical correlation on the prediction of correct equilibrium structure was rigorously detailed using the MCCI method in a large active space by augmenting the NCMET(ND) wave function. It was concluded that the most dominant double excitations were able to shift the minimum only about 0.5 Å, and the triple excitations play the crucial role in locating the correct minimum. The contribution of such triples also degrades at the dissociation limit. However, the most dominant triples alone were found insufficient, and all triple excitations accessible within the orbital basis set must be taken into account to predict the correct equilibrium structure. In this context, the SOCI and near-NCMET methods predicted the minimum at 2.45 Å, and successfully reproduced the binding energy at correct internuclear separation in excellent agreement with full CI predictions and the experiment.

SAPT calculations were also performed to clarify the covalent nature of the chemical bond in Be_2_, and the electrostatic and induction effects were found strong in Be_2_ molecule. Further calculations were carried out at MP6 and MP8 level of theories to investigate the contribution of dynamical correlation terms in detail. A benchmark-quality potential energy curve for the Be₂ molecule was obtained from full CI energy calculations at CBS limit. Accurate dispersion coefficients were predicted using full CI potential energy curve (C_6_ = 229 a.u., C_8_ = 10,659 a.u., C_10_ = 593,175 a.u.) which are consistent with prior estimates based on atomic polarizabilities. Furthermore, a new eight-parameter *modified Tang–Toennies model potential* was proposed to reproduce both the short-range repulsion and long range dispersion behavior of the molecule. The inclusion of the C_10_ term was found essential to support the full set of twelve vibrational states observed experimentally. These vibrational levels were computed from this model potential using the Lippmann–Schwinger equation solved in momentum space, yielding energy levels in better agreement with the experimental data.

In conclusion, this work provides a comprehensive analysis of the nature of the covalent bond in the beryllium molecule, highlighting the critical role of nondynamical and dynamical correlation effects and demonstrating the power of combining multireference methods and dispersion-corrected analytical models. The proposed methodology and parameters offer a solid reference for future theoretical and experimental studies on weakly bound diatomic systems with multireference character.

## Supplementary Information

Below is the link to the electronic supplementary material.

ESM 1 Supporting Information includes: Part A. CBS Extrapolation. Part B. Detailed Tabulation of Corrections Beyond The BO Approximation. Part C. The Details of The Calculation of The Scattering Length. Part D. Supplementary Discussion of Dynamical Correlation Effects. Part E. The List of The Determinants Included In NCMET(ND) Wave Function. (DOCX 170 KB)

## Data Availability

The data supporting the findings of this work are available in the Supporting Information file.
