# Supplementary material for: Multiconfigurational nature of the chemical bond in beryllium molecule
Source: J Mol Model. 2026 Jun 27;32(7):243. doi: 10.1007/s00894-026-06812-6 (PMC13310217; doi:10.1007/s00894-026-06812-6)
Supplement: Supplementary file 1 — Supporting Information includes: Part A. CBS Extrapolation. Part B. Detailed Tabulation of Corrections Beyond The BO Approximation. Part C. The Details of The Calculation of The Scattering Length. Part D. Supplementary Discussion of Dynamical Correlation Effects. Part E. The List of The Determinants Included In NCMET(ND) Wave Function. (DOCX 170 KB) [file 894_2026_6812_MOESM1_ESM.docx]

**SUPPORTING INFORMATION**

**Multiconfigurational Nature of The Chemical Bond In Beryllium Molecule**

Berkay Sütay

Istanbul Technical University, Department of Chemistry, 34469, Istanbul, Turkey

**A. CBS EXTRAPOLATION**

The following CBS extrapolation procedures were applied:

1. **Exponential extrapolation**: $E(n)=E_{CBS}+A\exp^{-Bn}$
2. **Power extrapolation**: $E(n)=E_{CBS}+An^{-\alpha}$
3. **Two-point X^-3^ formula**:

$$E_{CBS}=\frac{n^{3}E(n)-m^{3}E(m)}{n^{3}-m^{3}}$$

1. **Inverse power of angular momentum**:

$$E(n)=E_{CBS}+A\left(n+\frac{1}{2}\right)^{-4}$$

1. **Mixed Gaussian/Exponential extrapolation**: $E(n)=E_{CBS}+Ae^{-(n-1)}+Be^{-(n-1)^{2}}$

The spread of the resulting CBS limits was taken as an estimate of the extrapolation uncertainty. For this purpose, the CBS uncertainty was estimated as half of the maximum difference between the CBS limits obtained from different extrapolation schemes:

$$\Delta_{\text{CBS}} (\text{r})=\frac{\text{max}(\text{E}_{\text{CBS}} )-\text{min}(\text{E}_{\text{CBS}} )}{2}$$

**Table S1**. Full CI energies of ^∞^Be_2_ estimated using different CBS extrapolation procedures (a.u.).

r AVTZ AVQZ AV5Z CBS_exp CBS_power CBS_X^-3^ CBS_angular CBS_mixed uncertainty

2.0000 -29.22822799 -29.23015746 -29.23063627 -29.23079 -29.23092 -29.23113 -29.23116 -29.23091 0.00018

2.2000 -29.23733899 -29.23900545 -29.23944017 -29.23959 -29.23972 -29.23989 -29.23989 -29.23969 0.00015

2.4540 -29.24015482 -29.24158308 -29.24201182 -29.24219 -29.24235 -29.24245 -29.24238 -29.24226 0.00013

2.6000 -29.24005792 -29.24137887 -29.24175579 -29.24190 -29.24203 -29.24215 -29.24210 -29.24197 0.00012

2.8000 -29.23943159 -29.24064083 -29.24098905 -29.24112 -29.24124 -29.24135 -29.24130 -29.24119 0.00011

3.0000 -29.23883772 -29.23997289 -29.24029378 -29.24042 -29.24052 -29.24063 -29.24059 -29.24047 0.00010

3.2000 -29.23844056 -29.23952745 -29.23982666 -29.23994 -29.24003 -29.24014 -29.24011 -29.23999 0.00010

3.4000 -29.23820008 -29.23925572 -29.23953910 -29.23964 -29.23973 -29.23983 -29.23982 -29.23970 0.00009

3.6000 -29.23804394 -29.23907915 -29.23935135 -29.23944 -29.23953 -29.23963 -29.23963 -29.23950 0.00009

3.8000 -29.23792188 -29.23894324 -29.23921103 -29.23930 -29.23938 -29.23949 -29.23948 -29.23936 0.00009

4.0000 -29.23780966 -29.23882144 -29.23908051 -29.23916 -29.23924 -29.23935 -29.23935 -29.23923 0.00009

4.5000 -29.23754186 -29.23854103 -29.23879170 -29.23887 -29.23894 -29.23905 -29.23906 -29.23893 0.00009

5.0000 -29.23731900 -29.23831536 -29.23856171 -29.23864 -29.23871 -29.23882 -29.23883 -29.23870 0.00009

5.5000 -29.23716257 -29.23816076 -29.23840542 -29.23848 -29.23855 -29.23866 -29.23867 -29.23854 0.00009

6.0000 -29.23706420 -29.23806465 -29.23830863 -29.23838 -29.23845 -29.23856 -29.23858 -29.23844 0.00009

6.5000 -29.23700583 -29.23800763 -29.23825139 -29.23832 -29.23839 -29.23850 -29.23852 -29.23839 0.00009

7.0000 -29.23697178 -29.23797420 -29.23821804 -29.23829 -29.23836 -29.23847 -29.23849 -29.23835 0.00009

8.0000 -29.23693927 -29.23794248 -29.23818634 -29.23826 -29.23833 -29.23844 -29.23846 -29.23832 0.00009

9.0000 -29.23692572 -29.23793031 -29.23817414 -29.23825 -29.23831 -29.23842 -29.23844 -29.23831 0.00009

10.0000 -29.23691901 -29.23792497 -29.23816888 -29.23824 -29.23831 -29.23842 -29.23844 -29.23830 0.00009

50.0000 -29.23691054 -29.23791910 -29.23816321 -29.23824 -29.23830 -29.23842 -29.23844 -29.23830 0.00009


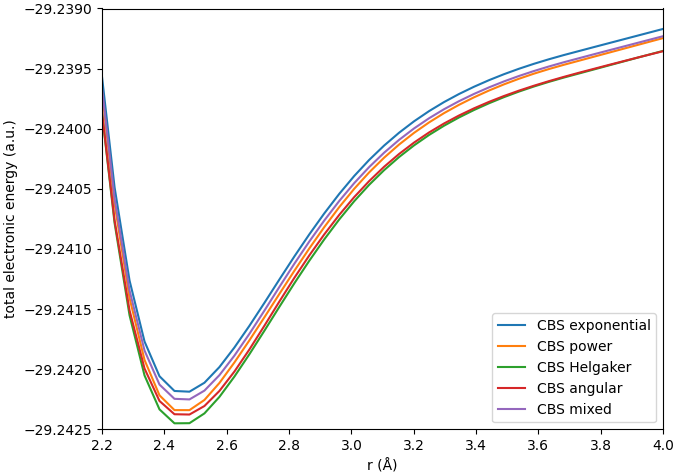


**Figure S1.** The CBS limit energies calculated using different extrapolation procedures.

**B. DETAILED TABULATION OF CORRECTIONS BEYOND THE BO APPROXIMATION**

NR all electron total electronic energy of ^∞^Be atom is -14.66736 [1, 2] and the experimental total energy is -14.66844 a.u. (calculated from NIST Database [3], up to the first uncertain decimal place).

**Table S2**. Relativistic and QED corrections to total electronic energy of beryllium molecule (a.u.).

|  | Be_2_ at R_e_ | Be_2_ at 8 Å | Δ (cm^-1^) |
| --- | --- | --- | --- |
| DBOC | +0.001841 | +0.001840 | +0.2 |
| Scalar relativistic  *Mass velocity:*  *1e-Darwin term:* | -0.00600  *-0.030060*  *+0.024060* | -0.00598  *-0.0300245*  *+0.0240450* | -4.4 |
| Breit terms | +0.0014067 | +0.0014049 | +0.4 |
| QED | +0.0007287 | +0.0007283 | +0.1 |

**C. THE DETAILS OF THE CALCULATION OF THE SCATTERING LENGTH**

The s-wave scattering length (a_s_) for Be + Be scattering was investigated by numerically solving the radial Schrödinger equation at the low-energy limit (k=0 Å^-1^). For that purpose, the radial wave function u(r) was integrated using Numerov algorithm, initiated from the classically forbidden region. A Neumann boundary condition (on the first derivative of the wave function) was imposed at the inner boundary to ensure physical consistency with the molecular core. The integration was extended into the asymptotic region, beyond 25 Å, where the V(r) potential becomes negligible and the wave function assumes its free-particle linear form: u(r) ~ (r - a_s_). The scattering length was then extracted via the logarithmic derivative matching or by identifying the intercept of the asymptotic linear trend with the r axis. The numerical integration yielded a scattering length about 1.25 Å. Within the attractive well of the potential, the radial wave function exhibited 12 nodes before entering the asymptotic regime, indicating the presence of 12 bound vibrational states, Figure S3. These results provide insight into the near-threshold bound state structure of the beryllium molecule.


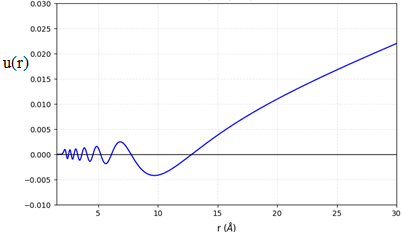


**Figure S2.** The scattering wave function at k=0.001 Å^-1^.

**D. SUPPLEMENTARY DISCUSSION ON DYNAMICAL CORRELATION EFFECTS**

Mk-MRCCSD method predicts the minimum at 4.5 Å due to the lack of triple excitations. The contribution of triple and quadruple excitations to the correlation energy were also estimated to be -0.0041 and -0.0068 a.u. respectively, and it is obvious that the quadruples also make a noticeable contribution to the valence correlation energy.


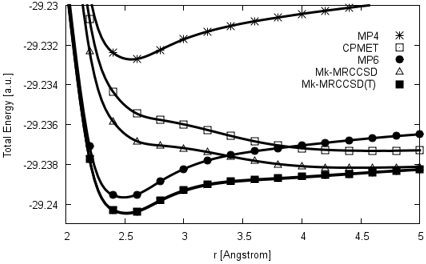


**Figure S3.** The PEC of ^∞^Be_2_ calculated by different level of theories in aug-cc-pVQZ basis.

An examination of the potential energy curves obtained using single-reference MPn methods, Figure S4, indicates that the MP6 method accounts for the effects of connected quadruple excitations (T_1_T_2_ cluster term is also included). The Mk-MRCCSD(T) approach also produces a smooth potential energy curve, however, it should be noted that the method is not invariant with respect to active orbital rotations [4]. The SOCI curve is almost identical to -slightly above- the MP6 curve. For beryllium molecule, the difference between the highly correlated MP6 and MP8 methods is the presence of T_1_^2^ and T_1_T_3_ cluster terms in MP8 level of theory. By considering the fact that the inclusion of new type of connected or disconnected correlation terms at even order of perturbation causes a coupling of these terms at the next odd order of perturbation. For that, MP9 energy may also be calculated over MP8 result. Beryllium molecule has four valence electrons, that is, MP9 is the highest level of perturbation method available in frozen-core approximation and T_1_^2^ and T_1_T_3_ terms are the only correlation effects arising at MP8 level. MP9 calculation in the same basis did not improve the electronic energy and produced identical binding energy with MP8.

**E. THE LIST OF THE DETERMINANTS INCLUDED IN NCMET(ND) WAVE FUNCTION**

The determinantal functions below are based on the following order of orbitals:

2σ_g_3σ_g_4σ_g_5σ_g_6σ_g_7σ_g_1π_ux_2π_ux_3π_ux_4π_ux_1π_uy_2π_uy_3π_uy_4π_uy_1δ2δ2σ_u_3σ_u_4σ_u_5σ_u_6σ_u_7σ_u_1π_gx_2π_gx_3π_gx_1π_gy_2π_gy_ 3π_gy_1ϕ2ϕ

**Be2 molecule NCMET(ND) WF**

200000000000000020000000000000

200000000000000020000000000000

220000000000000000000000000000

200000200000000000000000000000

200000000020000000000000000000

000000000020000020000000000000

000000200000000020000000000000

100000100000000010000010000000

100000000010000010000000010000

200000000000000000000020000000

200000000000000000000000020000

110000000000000011000000000000

020000000000000020000000000000

000000000000000022000000000000

000000000000000020000000020000

000000000000000020000020000000

200000000000000002000000000000

011000000000000020000000000000

110000000000000010100000000000

000000000011000020000000000000

000000110000000020000000000000

200000000011000000000000000000

200000110000000000000000000000

100100000000000011000000000000

211000000000000000000000000000

101000000000000011000000000000

100000000001000010000000010000

100000010000000010000010000000

000000000000000021010000000000

200000000000000001010000000000

010100000000000020000000000000

000000000000000020000011000000

000000000000000020000000011000

200000000000000000000000011000

200000000000000000000011000000

200000000000000001100000000000

000000000000000021100000000000

020000200000000000000000000000

020000000020000000000000000000

000000000020000000000000020000

000000200000000000000020000000

000000200020000000000000000000

020000000000000002000000000000

020000000000000000000000020000

020000000000000000000020000000

110000000000000000000000020000

110000000000000000000020000000

000000000020000000000020000000

000000200000000000000000020000

000000100010000000000010010000

110000000020000000000000000000

110000200000000000000000000000

010000000010000001000000010000

010000100000000001000010000000

000000000000000011000000020000

000000000000000011000020000000

000000000000000000000020020000

110000000011000000000000000000

110000110000000000000000000000

120100000000000000000000000000

110000000000000001100000000000

121000000000000000000000000000

000000110000000011000000000000

000000000011000011000000000000

020000000000000010010000000000

020000000000000010100000000000

020000110000000000000000000000

020000000011000000000000000000

110000000000000010010000000000

110000000000000010001000000000

110000000000000010000100000000

000000000010100020000000000000

000000000010010020000000000000

000000101000000020000000000000

000000100100000020000000000000

200000000010100000000000000000

200000000010010000000000000000

200000101000000000000000000000

200000100100000000000000000000

210100000000000000000000000000

210010000000000000000000000000

210001000000000000000000000000

100000000000100010000000010000

100000000000010010000000010000

100000001000000010000010000000

100000000100000010000010000000

000000000000000020000010100000

000000000000000020000000010100

200000000000000000000000010100

200000000000000000000010100000

000000000000000021001000000000

000000000000000021000100000000

010010000000000020000000000000

010001000000000020000000000000

100010000000000011000000000000

100001000000000011000000000000

200000000000000001001000000000

200000000000000001000100000000

110000000000000020000000000000

101000000000000020000000000000

100100000000000020000000000000

100010000000000020000000000000

100001000000000020000000000000

**References**

1. Chakravorty, SJ; Gwaltney, SR; Davidson, ER; Parpia, FA; Fischer, CF . Ground-state correlation energies for atomic ions with 3 to 18 electrons. *Phys Rev A*. 1993,47,3649-3670.

2. Hornyák, I; Adamowicz, L; Bubin, S . Ground and excited S 1 states of the beryllium atom. *Phys Rev A*. 2019,100,032504.

3. Kramida, A., Ralchenko, Yu., Reader, J. and [NIST ASD Team](https://www.physics.nist.gov/PhysRefData/ASD/index.html#Team) (2024). *NIST Atomic Spectra Database* (version 5.12), [Online]. Available: <https://physics.nist.gov/asd> [Mon Jan 12 2026]. National Institute of Standards and Technology, Gaithersburg, MD.

4. Lyakh DI, Musiał M, Lotrich VF, Bartlett RJ (2012) Multireference nature of chemistry: The coupled-cluster view. Chem. Rev. 112:182–243.
